# PVC/CNT Electrospun Composites: Morphology and Thermal and Impedance Behavior

**DOI:** 10.3390/polym16202867

**Published:** 2024-10-10

**Authors:** Marcio Briesemeister, John A. Gómez-Sánchez, Pedro Bertemes-Filho, Sérgio Henrique Pezzin

**Affiliations:** 1 Department of Materials Science and Engineering, Santa Catarina State University, Joinville 89219-710, Brazil; macio.brie@gmail.com; 2 CalipYuk Entreprises, Bogota 110110, Colombia; 3 Department of Electrical Engineering, Santa Catarina State University, Joinville 89219-710, Brazil; 4 Department of Chemistry, Santa Catarina State University, Joinville 89219-710, Brazil; sergio.pezzin@udesc.br

**Keywords:** carbon nanotubes, electrospinning, encapsulation, nanofibers, polyvinylchloride, impedance spectra

## Abstract

Due to their mechanical robustness and chemical resistance, composite electrospun membranes based on polyvinyl chloride (PVC) are suitable for sensor applications. Aiming to improve the electrical characteristics of these membranes, this work investigated the effects of the addition of carbon nanotubes (CNTs) to PVC electrospun membranes, in terms of morphology and thermal and impedance behavior. Transmission electron microscopy images evidenced that most of the nanotubes were encapsulated within the fibers and oriented along them, while field-emission scanning electron micrographs revealed that the membranes consisted of uniform fibers with an average diameter of 339 ± 31 nm, regardless of the addition of the carbon nanotubes. With respect to the neat resin, the addition of nanotubes caused a significant lowering of the glass transition temperature (up to 20 °C) and a marked change in the second degradation step of PVC. Nyquist plots from electrical impedance spectra showed a charge transfer resistance (R_CT_) of 38 and 40 MΩ for neat PVC and PVC/CNT 3 wt.% membranes, respectively, indicating that, in the dry state, the encapsulation of CNTs in the fibers and the high porosity of the membranes prevented the formation of a percolation network, increasing the electrical resistance. In the wet state, however, there was a greater change in the impedance behavior, decreasing the resistance R_CT_ to 4.5 and 1.1 MΩ, for neat PVC and PVC/CNT 3 wt.% membranes, respectively. The results of this study, showing a significant variation in impedance behavior between dry and wet membranes, are relevant for the development of various types of sensors based on PVC composites.

## 1. Introduction

Polyvinyl chloride (PVC) is one of the most consumed commodity polymers in the world, representing around 17% of the global market. It was one of the first polymers discovered and is currently undergoing full evolution [1,2,3]. The advancement in electrospinning technology observed in recent decades stands out among nanofiber preparation methods due to its advantages, including high controllability, simple operation, low cost, and wide adjustability [4]. These characteristics have led to expansion in the use of PVC for various applications: air filtration systems [5], water treatment, batteries, and protective clothing, among others. Due to the flexibility of the electrospinning process, it is possible to obtain nanofibers with diameters ranging from a few hundred nanometers to several micrometers. Among several interesting characteristics of electrospun PVC membranes, mechanical resistance, adjustable hydrophobicity, and high porosity can be highlighted [6].

Although the fibers obtained by the electrospinning technique have a high surface area, high porosity, and interesting electrochemical characteristics, nanofibers prepared with a single polymer often do not meet the requirements for practical applications. Due to its previously mentioned qualities, PVC is frequently employed in the manufacturing of nanocomposites, with the possibility of improving its mechanical properties, environmental resistance, etc. [1]. The application restrictions are mainly related to its high glass transition temperature (Tg), resulting from strong polar C-Cl interactions [2].

Composite electrospun fibers based on PVC can thus be used in many fields, including energy, sensors, and biomedicine, by the addition of other functional nanoparticles, such as carbon nanotubes (CNTs), during the electrospinning process [3,4]. The incorporation of carbon nanofillers to the PVC matrix can impact the mobility of the polymer chains and their effect on the structure depends on a variety of factors such as size, aspect ratio, and chemical functionalization [5]. Thus, the addition of CNTs to the PVC matrix promotes molecular mobility restrictions, due to the physical interaction between nanotubes and polymer molecules and the presence of an interphase between the matrix and the surface of the nanofiller, leading to changes in Tg and Tp (simultaneous flow temperature) [6]. For a well-dispersed system, a small number of nanotubes provides a large interfacial area, promoting changes in the mechanical, electrical, and thermal properties of the polymer [7,8].

The electrospinning of PVC membranes has been recently reviewed by Phan et al. [9] showing the huge potential of composite PVC nanomembranes. For instance, Namsaeng et al. [10], observed an increase of 127% and 175% in tensile strength and Young’s modulus, respectively, in electrospun PAN/PVC mats, evidencing a welding effect, and 205% and 314%, respectively, with the addition of 1 wt.% of MWCNTs. In another work investigating the addition of nanotubes, Elkasaby et al. [11] evaluated noise absorption in electrospun PVC membranes, observing that the addition of 5 wt.% CNTs improved sound absorption by 62% compared with the unfilled material, reaching a maximum value of the absorption coefficient of 0.4 at 800 Hz. Significant improvements in corrosion resistance in 6061T6 aluminum alloy with the use of electrospun PVC/ZnO nanofibers as a coating have been also reported [12].

Regarding electrical properties of electrospun membranes containing carbon nanotubes (CNTs), Yang et al. [13] reported that the addition of 0.05 wt.% CNTs to electrospun polyvinylidene fluoride (PVDF) nanofiber membranes provided highly sensitive flexible capacitive sensors. Sakamoto et al. [1] established a method to align CNTs on a substrate via an electrospinning process to maximize the intrinsic conductive and structural properties of CNTs. To this end, PEVA/CNT nanofibers were electrospun on an aluminum substrate and further heated in air to 400 °C to leave only the CNTs on the substrate. Impedance measurements showed that the charge transfer resistance was reduced from 20 Ω to 8 Ω with the alignment of the CNTs. Wang et al. [2] proposed that the electrochemical performance of fibers is largely determined by their structure, which is dependent on the parameters of the electrospinning process. The advantages of controllability in fiber morphology were highlighted, mentioning oriented, hollow, and porous fiber structures, which can improve the ion transport efficiency, promote intrinsic conductivity, and offer greater specific surface areas, providing a significant improvement in electrochemical performance.

Dror et al. [14] proposed a theoretical model to explain the behavior of particles such CNTs during the electrospinning process, which is related to the alignment of nanotubes in the process. The CNTs are initially randomly oriented, but because of the wedge-shaped flow, they become gradually oriented along the streamlines, so that as they approach the Taylor cone, the CNTs are aligned [15]. It is suggested that the alignment of the CNTs is also favored by the action of the electric field [16], where the intensity of the electric field, the exposure time, the concentration of the nanofiller and the viscosity of the solution are decisive. When the diameter of the fibers is reduced, the better the alignment of the nanotubes within the fibers, and irregularities in the coaxial alignment are reduced [4]. The reduction in fiber diameter prevents the random positioning of CNTs, making it difficult to form agglomerates. Therefore, it is essential to use an adequate concentration of CNTs. The high concentration tends to favor the formation of agglomerated regions, compromising the final properties of the composite. The results are also in line with Aliahmad et al. [15], who observed a unidirectional alignment of the CNTs inside the fibers due to the presence of an electric field acting on the nanotubes. This formation is suitable for many applications, covering sensors, reinforcements, and membranes.

However, no work related to the effect on the properties of electrospun PVC membranes of adding nanotubes has been reported to date. Therefore, the present study evaluated the influence of CNT addition on the morphology and the thermal and electrical behavior of electrospun PVC membranes, in order to understand the effect of adding CNTs to the membranes. This study can contribute to applied research in several areas, such as: air filtration [16,17], electrolytic sensors [18], potentiometric sensors [19], oil–water separation, energy storage [9,20], and metal ion removal from aqueous solutions [21]. The combination of high surface area (10,000 and 1,000,000 m^2^/kg for fibers with diameters of 500 and 5 nm, respectively) [22], flexibility (maintaining their properties after deformation) [9,22,23], and low production costs [9], can expand the field of use of a low-cost polymer in applications such as electrolytic sensors aimed at industry and health. This is the first work in a series that we intend to publish involving the application of electrospun PVC/CNT membranes in electrolytic sensors.

## 2. Materials and Methods

### 2.1. Materials

Homopolymer PVC (NORVIC^®^ SP 750RA), obtained by suspension polymerization, was supplied by Braskem (Camacari, Brazil). Multi-walled carbon nanotubes (MWCNTs) were purchased from Chengdu Organic Chemicals Co., Ltd. (Chengdu, China), batch TNIM4, with 95% purity. According to the manufacturer’s information, MWCNTs have an external diameter of 10–30 nm, an internal diameter of 5–10 nm, and a length of 10–30 μm.

Tetrahydrofuran (THF) and dimethylformamide (DMF), both supplied by Dinâmica (São Paulo, Brazil), with a minimum content of 99 and 99.8%, respectively, were used as solvents. Triton-X100, supplied by Vetec Química (Duque de Caxias, Brazil), was used as a surfactant.

### 2.2. Solution Preparation

PVC solutions were prepared in THF/DMF 50/50 (m/m) at a concentration of 18 wt.% containing amounts of 0%, 1%, 2%, and 3% by weight of CNTs. The detailed quantities of each component in the solutions are presented in Table 1.

**Figure 1:**
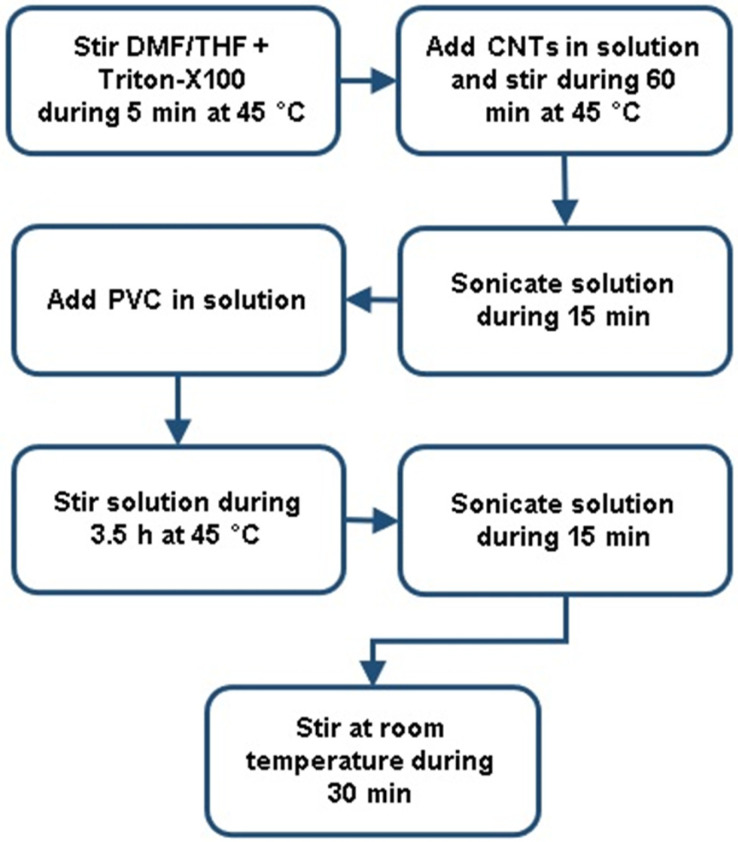
Scheme of the procedure for the preparation of PVC/CNT solutions.

The selection of different concentrations of CNTs in the electrospun PVC membranes was based on several existing studies in the literature, [4,24] as well as experimental tests with various other concentrations. During the membrane production process, it was observed that a CNT concentration higher than 3% hampered the electrospinning process. Figure 1 shows the procedure to produce the PVC/CNT solutions presented in Table 1.

### 2.3. Preparation of Electrospun Membranes

The PVC/CNT electrospun membranes were produced using Eletrotech Lab EF 2B CRT 0212 electrospinning equipment, manufactured by DBM Eletrotech (Joinville, Brazil). The equipment has an aluminum rotating collector with diameter of 60 mm and length of 200 mm, a high-voltage source for up to 20 kV, and two infusion pumps. Table 2 presents the settings used to produce the membranes.

**Figure 2:**
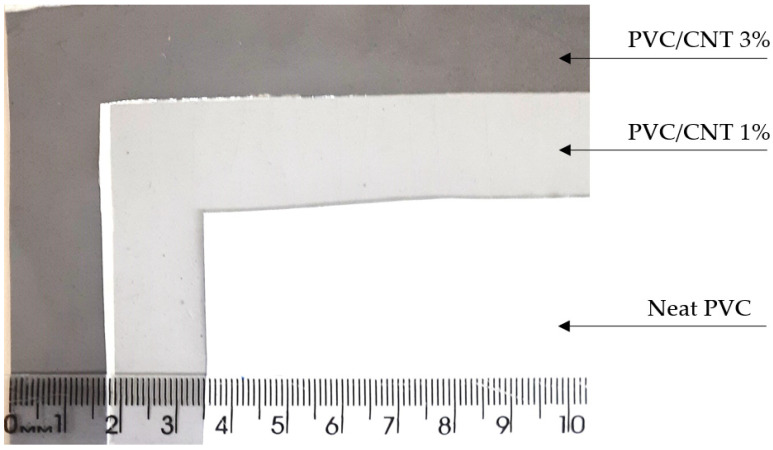
Physical image of the neat PVC, PVC/CNT 1 wt.%, and PVC/CNT 3 wt.% electrospun membranes.

A picture of the electrospun membranes is shown in Figure 2. The membranes were easily detached from the aluminum foil, with a usable size of approximately 15 × 15 cm (about 5.91 in) and a thickness of about 150–160 µm, which is directly dependent on the production time.

### 2.4. Thermal Characterization

Thermogravimetry (TG) was carried out in a TGA55 Analyzer (TA Instruments, New Castle, DE, USA). The heating rate was 10 °C/min, in a temperature range of 25 to 700 °C, under N_2_ atmosphere. The samples were ca. 5 mg.

Differential scanning calorimetry (DSC) analyses were carried out in DSC 200F3 equipment (Netzsch GmbH, Selb, Germany); the specimens were ca. 5 mg. The analyzes were run with heating and cooling rates of 10 °C/min, under N_2_ atmosphere, gas flow of 40 mL/min, and a closed panel system.

To obtain the first heating curves, the samples were heated from 25 °C to 165 °C, remaining at that temperature for 1 min. For the second heating, the material was cooled to 25 °C and subsequently heated again to 165 °C, remaining at that temperature for 1 min.

### 2.5. X-ray Diffractometry (XDR)

The XRD profiles of electrospun samples were measured in an XRD600 X-ray diffractometer (Shimadzu Scientific Instruments, Inc., Tokyo, Japan) at a wavelength of 1.5406 Å, 40 kV, and 30 mA. All samples were measured using an amorphous silicon sample holder, with 2θ varying from 10 to 70°, at 2°/min.

### 2.6. Morphological Characterization

Field-emission scanning electron microscopy (FESEM) was performed in a JSM-6701F (Jeol, Tokyo, Japan) operated at 10 kV. The samples were metallized with gold (Dentan Vacuum Desk V, Moorestown, NJ, USA) prior to the analyses.

Transmission electron microscopy (TEM) images were obtained with a JEM 2100 (Jeol) microscope. Samples were directly electrospun on carbon sample holders with 300 mesh copper grids (CF300-Cu).

### 2.7. Electrical Impedance Spectroscopy (EIS)

**Figure 3:**
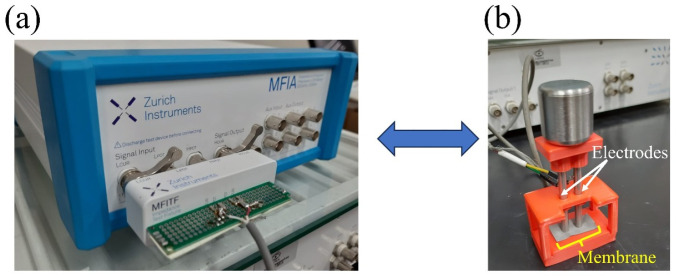
(**a**) Connection for electrical characterization using MFIA Zurich and (**b**) bipolar electrode for electrical measurements of membranes.

MFIA equipment (Zurich Instruments, Zurich, Switzerland) was used for the EIS analyses; the frequency range was between 10 Hz and 5 MHz, with 300 mV and current of 10 mA. The measurements were carried out using two stainless steel AISI 316 electrodes with polished contact surfaces, with a distance of 5 mm between the electrodes. The sample measurements were in the longitudinal direction of the orientation of the membrane fibers, each measurement was repeated five times. The membranes analyzed were between 150 and 160 μm thick. The samples were measured in the dry state and after being wetted with deionized water (Milli Q, Merck, Burlington, MA, USA). Figure 3 shows the device used in the measurements. The circuit simulation used Zview software version 2.8d.

## 3. Results and Discussion

**Figure 4:**
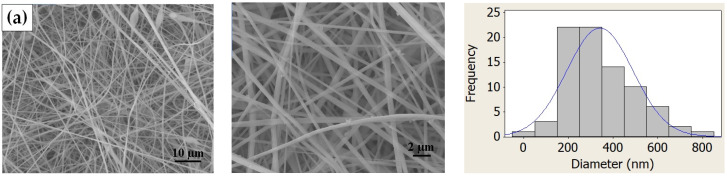

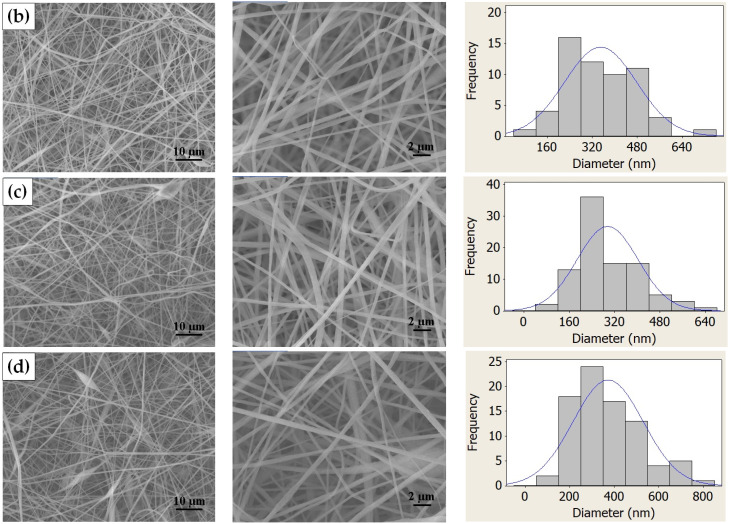
FESEM images of the electrospun membranes and fiber diameter histograms: (**a**) neat PVC, (**b**) PVC/CNT 1 wt.%, (**c**) PVC/CNT 2 wt.%, and (**d**) PVC/CNT 3 wt.%.

Figure 4 shows the FESEM images and fiber size distribution histograms of PVC electrospun membranes with different CNT concentrations.

**Figure 5:**
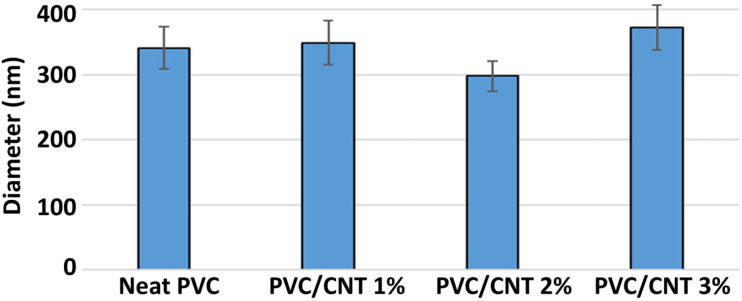
Diameter of electrospun PVC and PVC/CNT fibers.

It was observed that the membranes were composed of relatively uniform fibers containing few beads and flaws, except for the membranes containing 3 wt.% CNTs, in which there was a greater presence of beads next to the fibers. This was probably caused by the formation of CNT clusters in the solution, as also observed by Mazinani and Dubois for PET/CNT electrospun membranes [24]. The fibers presented average diameters of 295 to 373 nm, with significant differences only between the compositions containing 1 and 2 wt.%, and between 2 and 3 wt.%, with a significance of 95% considering the Mann–Whitney non-parametric hypothesis test. The average fiber diameters are shown in Figure 5.

Even though an increase in the concentration of CNTs in the solution can promote greater conductivity and a greater number of inductive charges, this did not result in a re-duction in the average diameter of the fibers. It is thus suggested that the concentration of CNTs was not sufficient for percolation to occur. The results are similar to those reported by Pham and Uspenskaya, who evaluated the influence of processing variables on the diameter of PVC nanofibers electrospun from 15 wt.% solutions at a flow rate of 1 mL/h [25].

Still regarding fiber diameter, no tendency towards reduction in fiber diameter was found due to the increase in the concentration of CNTs in the PVC matrix, unlike what was observed by Sharafkhani and Kokabi in electrospun PVDF/MWCNT fibers [4]. El Messiry and Fadel also observed a gradual reduction in the average fiber diameter when they increased the cellulose acetate (CA) concentration from 2% to 8% in PVC/CA bi-component nanofibers [5]. The addition of other nanoparticles to PVC, such as Fe, Fe_3_O_4_, Al, steel, and brass have also caused a decrease in the fiber diameters [6,7].

**Figure 6:**
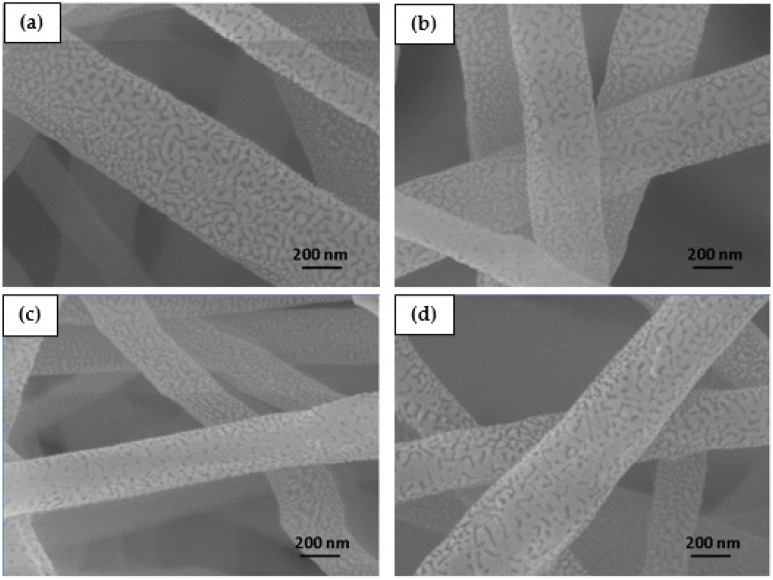
Surface roughness of electrospun blankets: (**a**) neat PVC, (**b**) PVC/CNT 1 wt.%, (**c**) PVC/CNT 2 wt.%, and (**d**) PVC/CNT 3 wt.%.

Figure 6 shows images demonstrating the surface roughness present in PVC/CNT fibers. It can be also noted that the roughness was independent of the CNT concentration. There are no studies in the literature reporting the surface morphology of PVC electrospun fibers obtained from THF/DMF solutions, but it is suggested that the formation of the rough structure presented on the surface of PVC/CNT fibers may be related to the mechanism described by Lin et. al. [26], who noted that when the molecular weight of PS diluted in THF/DMF is decreased, the entanglement of the polymer chains is reduced, accelerating the evaporation and diffusion of the solvent and, thus, causing a rapid phase separation on the surface and the creation of surface roughness.

Here, as different CNT concentrations did not affect the surface morphology of the fibers, it is suggested that the concentration of the nanofiller did not impact the solvent evaporation process.

**Figure 7:**
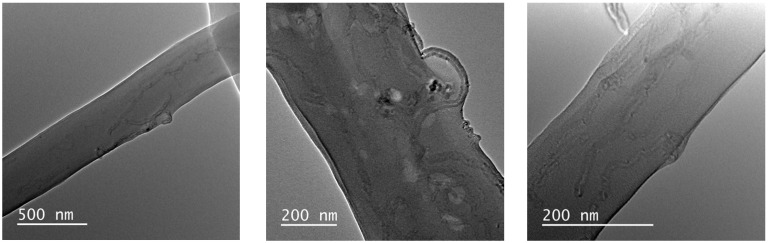
Dispersion of 3% CNTs inside electrospun PVC.

TEM images presenting the dispersion of CNTs inside electrospun PVC fibers are shown in Figure 7.

Good adhesion was observed between the CNTs and the polymer matrix, with only a few regions presenting agglomerates. Most of the nanotubes were located inside the fibers and oriented along them, i.e., encapsulated, and only a small portion were positioned on the external side of the fibers. The CNTs that were not fully encapsulated in the PVC fibers was due to their non-uniformity, presenting folds, and favoring mechanical entanglement [27]. It is suggested that the alignment of the nanotubes can be explained by the combination of the wedge-shaped model [14], the action of the electric field on the nanotubes [28], and the fiber diameter [4], as described in Section 1.

**Figure 8:**
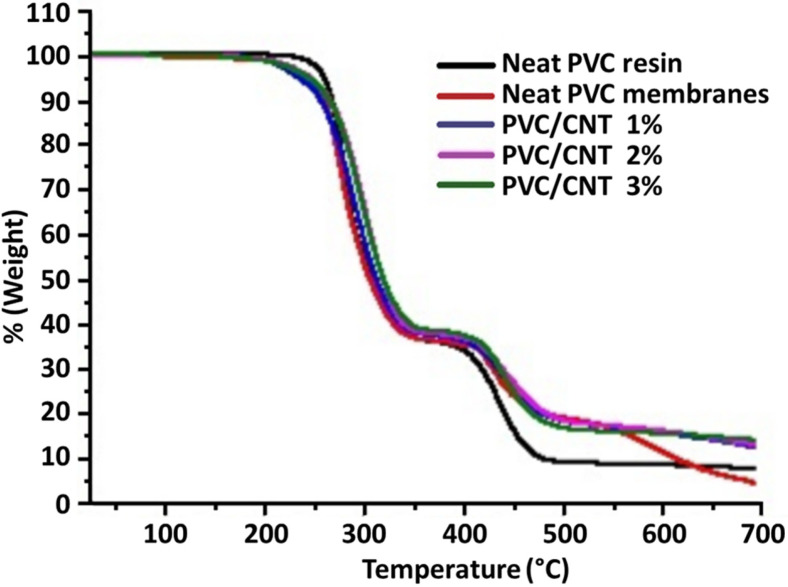
Thermogravimetric curves of electrospun PVC/CNT membranes and neat SP750Ra resin.

The consequence of the addition of nanotubes on thermal behavior was evaluated by TG and DSC. Figure 8 presents the mass loss profiles of neat PVC and PVC/CNT electrospun membranes.

A small mass loss was observed below 150 °C in the PVC/CNT membranes, which can be attributed to the vaporization of THF and DMF that were retained in the PVC matrix after electrospinning. Both resin and PVC/CNT nanocomposites presented two stages of degradation. In the first stage, attributed to the PVC dehydrochlorination reaction, a mass loss of 64% was observed for the resin and 62% for the fibers containing 3 wt.% CNTs, where the mass loss began shortly after 200 °C. For the other PVC/CNT compositions, the mass loss was intermediate between the two values. In the second stage, a mass loss of 17% and 11% was observed for the resin and the nanocomposite, respectively, where the mass loss began at around 400 °C, being attributed to the rupture of the double bonds of the polyene structure, forming volatile hydrocarbons in addition to solid char residue. From 550 °C onwards, variations in the TG curves are observed, which can be attributed to the different concentrations of CNTs in the structure. Analyzing the results, although the presence of CNTs did not affect significantly the first degradation step, there was a marked change in the second degradation step of PVC, indicating that the presence of nanotubes favored the formation of a greater amount of residue (char). The TG curves are also similar to those found in the study by Hasan et. al., who also evaluated PVC/CNT fibers [29].

**Figure 9:**
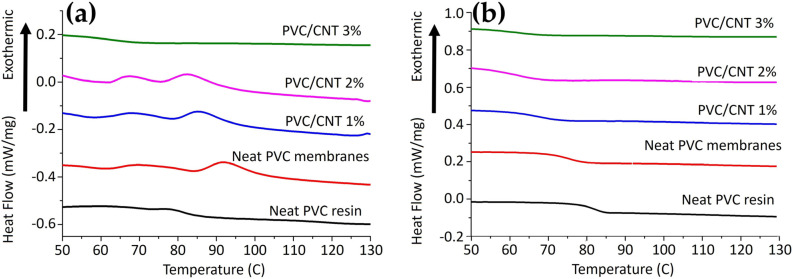
DSC curves of SP750Ra resin and PVC/CNT membranes: (**a**) first heating and (**b**) second heating.

Figure 9 shows the DSC curves for the first and second heating of PVC/CNT membranes and the neat PVC resin.

The results show a well-defined trend in the reduction in the glass transition temperature (Tg) as the concentration of CNTs in the composite increases. Table 3 presents the detailed results.

It is worth noting that, in the first heating (Figure 6a), just above Tg, an endothermic hysteresis peak is observed, characteristic of volume relaxation due to the rapid formation of the fiber structure during the electrospinning process, as observed by Ero-Phillips et al. [30] and Bognitzki et al. [31] for PLLA fibers.

The variation in the Tg is strongly related to the mobility of the PVC polymer chains, which can be influenced by the molecular mass and tacticity and the interaction of the nanotubes and the matrix [32]. Nevertheless, the results do not agree with several other studies reviewed by Tomaszewska et al. [32], which reported that the addition of CNTs to PVC matrices provided an increase in Tg, by reducing the mobility of the polymer chains. It is suggested that this may be associated with the saturation limit and dispersion of nanotubes in the matrix, causing a plasticizing effect on the composite. Agglomerates formed at high CNT concentrations may also have relatively poor interfacial interactions with the matrix.

**Figure 10:**
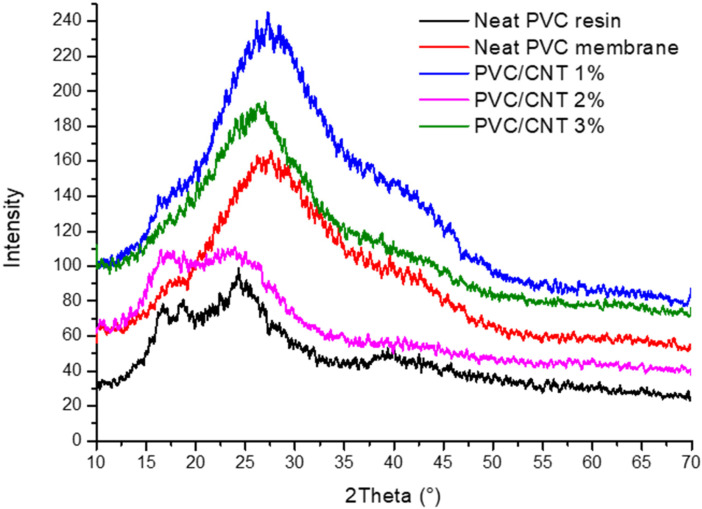
X-ray diffraction profiles for neat PVC resin (SP750Ra), neat PVC electrospun membranes, and PVC electrospun membranes containing 1%, 2%, and 3% CNTs.

The X-ray diffraction profiles of the electrospun samples, as well as the PVC resin, are shown in Figure 10.

The XRD profile for the virgin PVC resin shows a halo between 2θ 15 and 30°, indicating the mostly amorphous nature of the material, with discrete typical peaks at 16.5°, 18.5°, and 24°, related, respectively, to the (200), (110), and (201) planes of the orthorhombic PVC unit cell [33,34]. Regarding the electrospun samples, for the neat PVC membrane, the amorphous halo is observed between 19° and 35°, with no indication of crystalline peaks, suggesting that the solvent evaporation process occurred quickly, modifying the original structure of the material [35]. In samples containing CNT, as the concentration of nanotubes in the matrix was increased, the discrete peaks at 2θ 16.5° and 18.5° were suppressed, showing that CNTs hindered the formation of crystallites. Specifically for the PVC/CNT 2% membrane, a profile like that found for the virgin (unprocessed) PVC resin can be observed, which may be related to the non-uniformity in the dispersion of nanotubes in the matrix for this sample [36].

**Figure 11:**
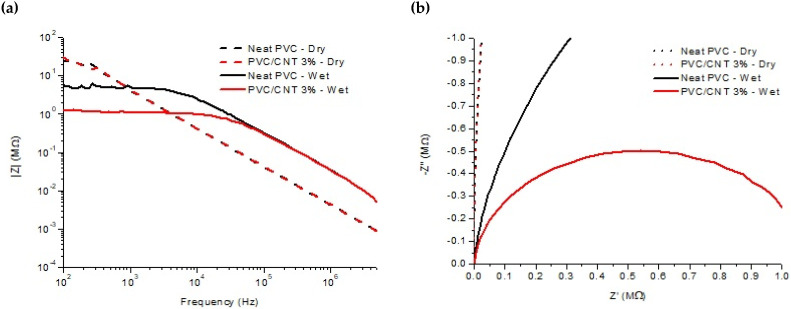
Electrical impedance spectroscopy: (**a**) Bode plot and (**b**) Nyquist plot.

Figure 11 presents the results of electrical impedance spectroscopy of pure PVC and 3% PVC/CNT electrospun membranes. Analyzing the results of |Z| (Figure 11a), no significant differences were found between the two samples considering the non-parametric Mann–Whitney test for a significance of 95%. The impedance spectra (Bode plot) of dry membrane samples show a typical insulating behavior, with slopes near −1 [37].

**Figure 12:**
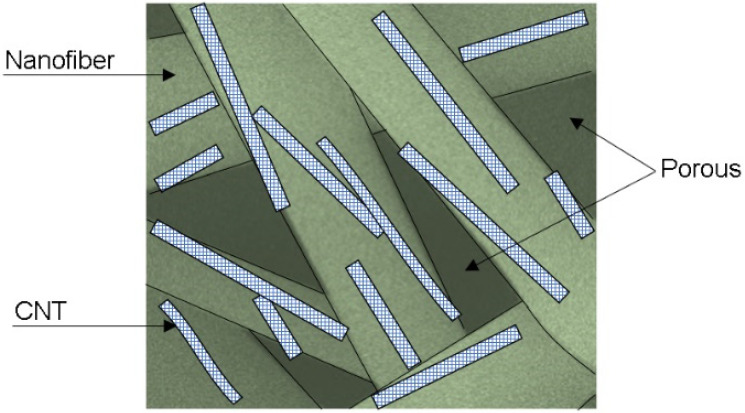
Schematic illustration of a PVC/CNT electrospun membrane.

Figure 11b shows Nyquist plots for dry and wet samples of electrospun PVC and PVC/CNT 3% membranes. From the measurements in the dry state, charge transfer resistances (R_CT_) of 38 MΩ and 40 MΩ were estimated for neat and composite PVC membranes, respectively. The results confirm the resistive characteristic of the material regardless of the presence of CNTs in the structure, i.e., the addition of 3 wt.% CNTs did not interfere with the impedance spectroscopy results. It is suggested that the reason for non-percolation was basically due to two factors: the first was the encapsulation of the vast majority of CNTs within the fibers and the second was the high porosity of the membranes caused by the spaces (voids) between the fibers. Figure 12 schematically illustrates the CNTs encapsulated inside the fibers and the presence of pores or spaces between them.

Thus, despite the high electrical conductivity of CNTs, the PVC/CNT 3% membrane, in the dry state, presented almost the same electrical behavior as the insulating porous PVC matrix, as there was no formation of a conducting network due to the encapsulation of the nanotubes within the fibers. The pores, within and between the fibers, also played an important role in determining the physical and chemical properties of electrospun membranes [38].

A significant reduction in R_CT_ was observed for the membranes wetted with deionized water, to approximately 4.5 and 1.1 MΩ, for the neat PVC and PVC/CNT 3% membranes, respectively. For the neat PVC membrane, it is suggested that the electrical conductivity was a result of the Grotthuss mechanism. Meanwhile, in the composite produced with PVC/CNT 3%, it is suggested that the electrical conductivity occurred through a combination of ionic and electronic conductivity [19]. This difference in the impedance behavior of dry and wet membranes, showing a significant variation of R_CT_, can lead to the development of various types of flexible ionic sensors based on PVC composites.

Table 4 below summarizes the results found and compares them with those found in the literature.

## 4. Conclusions

Aiming to develop robust materials for sensors PVC, electrospun membranes were prepared with the addition of carbon nanotubes. Electrospinning processing limited the concentration of nanotubes in the composites to 3%. Most of the nanotubes were encapsulated and oriented longitudinally relative to the fibers, with a small portion positioned externally. Furthermore, the addition of nanotubes did not affect the surface morphology nor the average diameter (approximately 339 ± 31 nm) of the fibers. A significant reduction (from 82 to 62 °C) was noted in the glass transition temperature of the composite containing 3% CNTs, in comparison with neat PVC, which can be related to a saturation of the nanofiller in the matrix, hindering its dispersion and ultimately resulting in greater mobility of polymer chains. With respect to the results of electrical impedance spectroscopy, no significant differences were found due to the presence of CNTs in dry PVC membranes, with high resistivity related to the porosity and the encapsulation of the CNTs within the fibers, which prevented the formation of a percolation network in the composite. A significant reduction in the charge transfer resistance (ca. 97% for composite membranes containing 3 wt.% CNTs) was observed for the membranes wetted with deionized water. This difference in the impedance behavior can lead to the development of various types of flexible ionic sensors based on PVC composites. To better understand the effect of encapsulated CNTs in electrospun PVC nanofibers, however, further investigations into the capacitive behavior of the material are needed to determine energy storage efficiency, as well as its stability and durability over multiple charge and discharge cycles.

## Figures and Tables

**Table 1:** Composition of the PVC/CNT solutions (in 20 mL THF/DMF 50/50).

Name	PVC (g)	CNTs (g)	Triton X-100 (g)
**Neat PVC**	3.6	-	-
**PVC/CNT 1%**	3.6	0.036	0.118
**PVC/CNT 2%**	3.6	0.072	0.118
**PVC/CNT 3%**	3.6	0.108	0.119

**Table 2:** Parameters used for the electrospinning processes.

Variables	Values
**Flow rate**	3 mL/h
**Work distance**	15 cm
**Collector rotation**	160 rpm
**Syringe**	10 mL
**Needle**	Ø0.7 × 30 mm
**Temperature**	23 °C
**Humidity**	42–55%

**Table 3:** Tg values from DSC curves of SP750Ra resin and PVC/CNT membranes.

Name	1st Heating (°C)	2nd Heating (°C)
**Neat PVC resin**	82	82
**Neat PVC membrane**	79	75
**PVC/CNT 1%**	74	66
**PVC/CNT 2%**	71	62
**PVC/CNT 3%**	62	61

**Table 4:** Summary of the results of this work and those reported in the literature for similar materials.

Properties	Results	Literature	References
Morphological	Fiber diameter = 339 ± 31 nm	Fiber diameter = 1008 ± 137 to 388 ± 84, respectively, for neat PET to PET/CNT 5% *w*/*w*	[24]
		Fiber diameter = 338 nm (PVC 15 wt.%, 0.5 mL/h, 20 KV, 15 cm and THF/DMF 50/50) in neat PVC	[25]
		Fiber diameter = 197 ± 15 to 90 ± 6, respectively, for neat PVDF to PVDF/CNT 1.5 wt.%	[4]
	CNTs encapsulated and aligned	CNTs aligned with aggregate formation	[24]
		CNTs aligned with fibers	[4]
	PVC fiber surface roughness	PS fiber surface roughness	[26]
	Amorphous nature (X-ray)	Amorphous nature (X-ray)	[33,34,36]
Thermal	Degradation curve (TGA)	PVC/CNT fibers	[29]
		PVC/CNT films	[39]
	Glass transition (Tg): 82 to 62 °C, respectively, for neat PVC resin and PVC/CNT 3 wt.%	Glass transition (Tg): 69 to 70.5 °C, respectively, for PVC/CNT 0.01 wt.% and PVC/CNT 0.05 wt.%	[32]
		Glass transition (Tg): 79 to 116 °C, respectively, for neat PVC and PVC/CNT 0.5 wt.%	[40]
		Glass transition (Tg): 79 to 119 °C, respectively, for neat PVC and PVC/CNT 0.5 wt.% (phosphorylated)	[40]
Impedance spectra	38 MΩ and 40 MΩ, respectively, for pure PVC and PVC/CNT 3 wt.%	Microfibers with high CNT concentrations: 110 MΩ to 22 MΩ, respectively, for agarose/CNT (5% RH) and agarose/CNT (98% RH)	[18]
		Microfibers with low CNT concentrations: 3.5 KΩ to 58 KΩ, respectively, for agarose/CNT (5% RH) and agarose/CNT (98% RH)	
		PVC/CNT films: percolation concentration threshold from 0.5 wt.% CNTs	[41]
		PVC films: 61 MΩ to 25 MΩ, respectively, for neat PVC and PVC with 10 wt.% NH4I	[37]

## Data Availability

Data are contained within the article.
